# Supplementary material for: Sequence-Based Viscosity Prediction for Rapid Antibody Engineering
Source: Biomolecules. 2024 May 23;14(6):617. doi: 10.3390/biom14060617 (PMC11202045; doi:10.3390/biom14060617)
Supplement: Supplementary file 1 [file biomolecules-14-00617-s001.zip › biomolecules-2997693-supplementary.pdf]

Supplementary Table S1

Schrodinger Surface Patch Analysis of Prominent Patches of MMAb3 from PDB:7REW after removal of IL-13. Patches of smaller size or patch score were excluded. For negatively charged patch 44, surface analysis revealed residues with the highest contribution in kcal/mol were most prevalent in the light chain. Residues lacking a negative charge generally had a significantly lower contribution score.

Patch ID	Size (Å²)	Patch Score	Residue (ref)	Topo	Contribution (kcal/mol)	Side Chain Accessibility
44	614	776.47	**LC:E3**	**LC:FR1**	**116.07**	**57.0%**
			**LC:D26**	**LC:CDR1**	**100.87**	**58.2%**
			**LC:D33**	**LC:CDR1**	**100.77**	**47.8%**
			**LC:D87**	**LC:FR3**	**82.47**	**50.7%**
			**HC:E53**	**HC:FR2**	**48.09**	**39.1%**
			**LC:Y2**	**LC:FR1**	**46.86**	**51.7%**
			**LC:D67**	**LC:CDR2**	**41.44**	**26.3%**
			LC:S111	LC:CDR3	28.28	63.4%
			LC:G32	LC:CDR1	25.77	21.4%
			LC:K30	LC:CDR1	22.34	41.2%
			LC:Y40	LC:CDR1	21.72	40.0%
			LC:T5	LC:FR1	20.59	48.2%
			**LC:D110**	**LC:CDR3**	**15.46**	**18.9%**
			LC:N82	LC:FR3	13.37	6.1%
			HC:E99	HC:FR3	12.67	49.0%
			LC:T135	LC:CDR3	12.58	34.0%
			LC:F139	LC:FR4	10.98	7.4%
			LC:K39	LC:CDR1	9.25	19.3%
			HC:L52	HC:FR2	8.94	0.3%
			LC:S24	LC:CDR1	8.35	46.2%
			LC:G84	LC:FR3	7.6	108.3%
			LC:S1	LC:FR1	6.01	34.8%
			LC:H58	LC:CDR2	5.77	22.7%
			LC:V138	LC:CDR3	5.16	20.0%
9	512	542.25	**HC:K86**	**HC:FR3**	**112.73**	**47.4%**
			**HC:R20**	**HC:FR1**	**97.49**	**41.6%**
			**HC:R97**	**HC:FR3**	**89.75**	**39.2%**
			HC:N94	HC:FR3	25.48	40.9%
			HC:R77	HC:FR3	23.89	16.6%
			HC:S7	HC:FR1	18.78	52.6%
			HC:T144	HC:FR4	18.78	52.1%
			HC:G8	HC:FR1	17.52	95.6%
			HC:S73	HC:FR3	17.01	41.9%
			HC:S95	HC:FR3	16.93	55.4%
			HC:Y90	HC:FR3	16.56	19.2%
			HC:S149	HC:FR4	16.33	56.3%
			HC:Q92	HC:FR3	13.79	33.9%
			HC:T143	HC:FR4	11.89	23.1%
			HC:S81	HC:FR3	9.28	44.1%
			HC:D72	HC:FR3	6.96	57.7%
			HC:P15	HC:FR1	6.65	28.6%
			HC:G10	HC:FR1	6.54	30.1%
			HC:S18	HC:FR1	5.78	42.9%
			HC:S22	HC:FR1	5.06	34.5%
			HC:A98	HC:FR3	5.03	59.3%
